# Long-Term Sinonasal Carriage of *Staphylococcus aureus* and Anti-Staphylococcal Humoral Immune Response in Patients with Chronic Rhinosinusitis

**DOI:** 10.3390/microorganisms9020256

**Published:** 2021-01-27

**Authors:** Ulrica Thunberg, Svante Hugosson, Ralf Ehricht, Stefan Monecke, Elke Müller, Yang Cao, Marc Stegger, Bo Söderquist

**Affiliations:** 1 Department of Otorhinolaryngology, Örebro University Hospital, SE 70185 Örebro, Sweden; svante.hugosson@regionorebrolan.se; 2 Faculty of Medicine and Health, Örebro University, SE 70182 Örebro, Sweden; MTG@ssi.dk (M.S.); Bo.Soderquist@oru.se (B.S.); 3 InfectoGnostics Research Campus, 07743 Jena, Germany; Ralf.Ehricht@leibniz-ipht.de (R.E.); stefan.monecke@leibniz-ipht.de (S.M.); Elke.Mueller@leibniz-ipht.de (E.M.); 4 Leibniz Institute of Photonic Technology (IPHT), 07743 Jena, Germany; 5 Institut fuer Medizinische Mikrobiologie und Hygiene, Medizinische Fakultaet “Carl Gustav Carus” Fiedlerstr. 42, D-01307 Dresden, Germany; 6 Institute of Physical Chemistry, Friedrich-Schiller University, 07743 Jena, Germany; 7 Clinical Epidemiology and Biostatistics, School of Medical Sciences, Örebro University, SE 70182 Örebro, Sweden; yang.cao@regionorebrolan.se; 8 Department of Bacteria, Parasites and Fungi, Statens Serum Institut, 2300 Copenhagen, Denmark; 9 Department of Laboratory Medicine, Clinical Microbiology, Örebro University Hospital, SE 70185 Örebro, Sweden

**Keywords:** chronic rhinosinusitis, immunoglobulins, *Staphylococcus aureus*, whole-genome sequencing, enterotoxin, long-term, carriage, antigen

## Abstract

We investigated *Staphylococcus aureus* diversity, genetic factors, and humoral immune responses against antigens via genome analysis of *S. aureus* isolates from chronic rhinosinusitis (CRS) patients in a long-term follow-up. Of the 42 patients who provided *S. aureus* isolates and serum for a previous study, 34 could be included for follow-up after a decade. Clinical examinations were performed and bacterial samples were collected from the maxillary sinus and nares. *S. aureus* isolates were characterized by whole-genome sequencing, and specific anti-staphylococcal IgG in serum was determined using protein arrays*. S. aureus* was detected in the nares and/or maxillary sinus at both initial inclusion and follow-up in 15 of the 34 respondents (44%). Three of these (20%) had *S. aureus* isolates from the same genetic lineage as at inclusion. A low number of single-nucleotide polymorphisms (SNPs) were identified when comparing isolates from nares and maxillary sinus collected at the same time point. The overall change of antibody responses to staphylococcal antigens over time showed great variability, and no correlation was found between the presence of genes encoding antigens and the corresponding anti-staphylococcal IgG in serum; thus our findings did not support a role, in CRS, of the specific *S. aureus* antigens investigated.

## 1. Introduction

Chronic rhinosinusitis (CRS) affects about 10% of the European population, and is a burdensome disease in which *Staphylococcus aureus* is suggested to have a potential role [1]. The presence of *S. aureus* in healthy maxillary sinuses is low [2], but it is commonly observed in patients with CRS [3,4,5]. *S. aureus* is generally considered to be a harmless commensal which colonizes the nares intermittently or persistently in 20–70% of healthy individuals [6,7,8]. However, it also has the potential to cause invasive diseases ranging from mild superficial skin disorders such as folliculitis to serious conditions including sepsis and infective endocarditis [9]. *S. aureus* superantigens has been shown to contribute to inflammatory diseases such as rheumatoid arthritis, asthma, and atopic dermatitis [10,11,12,13], and are suggested to have an impact on CRS [14,15,16]. The nares are regarded as the most important colonization site, since eradication from the nares can result in subsequent disappearance of *S. aureus* from other parts of the body [7].

Important factors related to pathogenicity include staphylococcal enterotoxins, enzymes, and cell-surface-associated virulence components [17,18,19]. Staphylococcal superantigens, such as enterotoxins, have been shown to enhance mucosal eosinophilia by Th-2 cytokine release and further T cell actions in CRS with nasal polyposis [20]. *S. aureus* within biofilm retains its capacity to release toxins, which can promote the inflammatory response in CRS [21,22]. Levels of IgG antibody against toxic shock syndrome toxin-1 (TSST-1) and LukF-PV, a subunit of the Panton–Valentine leukocidin (PVL), has shown to be significantly higher in CRS patients than in healthy controls, which could indicate that these specific *S. aureus* antigens affect the pathogenesis of CRS disease [23].

Further insight into microbial and host-specific factors could be of importance for the understanding of CRS, including the role of *S. aureus* in the pathogenesis [1]. Long-term studies of the anti-staphylococcal immune response in CRS patients and genetic characterization of *S. aureus* in the sinonasal cavities in cohorts of CRS patients are rare. We therefore aimed to study the persistence of S. aureus in the nares and maxillary sinus of CRS patients by performing genomic analyses of *S. aureus* and by determining serum antibody responses to specific staphylococcal antigens in a long-term follow-up of a Swedish cohort of patients with CRS.

## 2. Materials and Methods

### 2.1. Collection of Serum and Bacterial Samples from Patients with CRS

Primary inclusion took place as part of a previous study performed in 2004–2010 [4]. Subjects were of age >18 years and were diagnosed with CRS by an otorhinolaryngology (ORL) specialist. Two physicians specialized in ORL (authors UT and SH) performed all inclusion procedures. All patients had had surgery when the collection of specimens was performed. No patients were immunocompromised or had on-going antibiotic treatment at time for inclusion. Three patients had short course oral corticosteroid treatment at time of inclusion. *S. aureus* was isolated from the maxillary sinuses of 18/42 CRS patients (43%) and the nares of 24/42 CRS patients (57%), and serum samples from 29/42 CRS patients were collected and stored. The same cohort of patients, aside from two who were deceased, was contacted between 2017 and 2019 for a follow-up study. The first data collection is referred to as time point C1 and the second as C2. Bacterial samples were collected from the maxillary sinus and nares at C2 using the same protocol as at C1. Blood samples were collected from patients at C1 and sera were stored at −80 °C pending analyses. Additional blood samples were collected at C2. Sampling techniques and handling of specimens have been described previously [4]. Culturing and species verification of *S. aureus* were performed in accordance with routine diagnostic procedures at the Department of Laboratory Medicine, Clinical Microbiology, Örebro University Hospital. All isolates were stored at −80 °C in preservation medium (trypticase soy broth, BD Diagnostic Systems, Sparks, MD, USA) supplemented with 0.3% yeast extract (BD Diagnostic Systems) and 29% horse serum (SVA, Uppsala, Sweden). Thirty-four of the 40 patients from the cohort agreed to be included in the follow-up study.

### 2.2. Antibiotic Susceptibility Testing

*S. aureus* isolates obtained at C1 and C2 were suspended in sterile saline to 0.5 McFarland. Susceptibility testing was performed on Mueller-Hinton II agar 3.8% *w*/*v* (BD Diagnostic Systems, Sparks, MD, USA) using the standardized disk diffusion method in accordance with the European Committee on Antimicrobial Susceptibility Testing (www.eucast.org) guidelines for the following antibiotics: cefoxitin (30 µg), fusidic acid (10 µg), erythromycin (15 µg), clindamycin (2 µg), rifampicin (5 µg), gentamicin (10 µg), trimethoprim-sulfamethoxazole (25 µg), tetracycline (30 µg), and norfloxacin (10 µg). All disks were from Oxoid, Basingstoke, UK.

### 2.3. DNA Sequencing and Single-Nucleotide Polymorphism (SNP) Analysis

Whole-genome sequencing (WGS) of *S. aureus* was performed on genomic DNA extracted using the QIAGEN DNeasy Blood and Tissue Kit (QIAGEN, Hilden, Germany), with subsequent library construction using the Nextera XT Kit (Illumina, Little Chesterford, UK); specifically, a 300-cycle kit on the NextSeq platform (Illumina) according to the manufacturer’s instructions. The genomes were assembled using SPAdes v3.10.1 and MLST typed with the MLST command-line tool (https://github.com/tseemann/mlst).

A SNP-based phylogeny was created to assess the relationship between C1 and C2 isolates, using SNPs identified using NASP [24] with the BWA algorithm to align Illumina reads from individual isolates against the chromosome from the ST45 *S. aureus* isolate CA-347 (GenBank accession number: CP006044) [25] after removal of duplicated regions using NUCmer. Positions with less than 10-fold coverage and less than 90% unambiguous variant calls were excluded. A midpoint rooted maximum-likelihood phylogenetic tree was calculated in IQ-TREE [26].

For determining the presence of key genes in the assembled genomes, the QIAGEN CL Genomics Workbench v12.0 (QIAGEN, Aarhus, Denmark) was used to perform a BLASTN search with positive hits required to have >75% hit length and >85% sequence similarity.

### 2.4. Microarray-Based Immunoglobulin Analysis

The method regarding protein microarrays was previously described by Kloppot et al. [27] and Selle et al. [28] and from that source information about the antigens could be extracted. The antibody specificity patterns of the paired serum samples from C1 and C2 were analyzed using a protein microarray-based assay comprising 61 different *S. aureus-*specific antigens (Alere Technologies GmbH, Jena, Germany). All purified proteins were covalently immobilized to the array surface as duplicates using different concentrations ranging from 0.01 to 0.5 mg/mL. Purified IgG antibodies from different species (human, bovine, and murine) and one HRP-labeled protein served as positive controls.

Antibody detection using these protein microarrays was performed according to the following protocol. The microarrays were first incubated twice with washing buffer (1 × PBS/0.05%, Tween 20/0.25%, and TritonX100) at 37 °C and 400 rpm for 3 min. Next, they were incubated with blocking buffer (1 × PBS/0.05%, Tween 20/0.25%, TritonX100, and 2% milk powder) at 37 °C and 300 rpm for 5 min. The diluted serum samples (1:100) were incubated for 30 min at 37 °C and 300 rpm. After a washing step as described above (37 °C, 400 rpm, 5 min), the microarrays were incubated with a diluted (1:1000) HRP-labeled anti-human IgG-HPR antibody (Sigma-Aldrich, Taufkirchen, Germany) at 37 °C and 300 rpm for 30 min. The protein arrays were then washed twice with washing buffer (37 °C, 400 rpm, 3 min). Finally, the arrays were incubated with the substrate Seramun Green (Seramun, Heidesee, Germany) for 10 min without shaking at room temperature. The protein arrays were read out with the ArrayMate, and the data were analyzed using IconoClust software according to the manufacturer’s specifications (Alere Technologies GmbH, Jena, Germany).

Relative signal intensities of defined regions (at predefined spot coordinates) on the Staph-Toxin-Array were determined during readout. The signal intensities of the individual spots were normalized (NI). For this purpose, the average intensities were first calculated from all valid pixels of the local background (BG) and all valid pixels of the spot (M). The normalization of the spot intensity against the local background was then performed using the formula NI = 1 − (M/BG). Therefore, the NI values were between the value 0 (undetectable signal) and 1 (maximum signal).

### 2.5. Statistical Analysis

The Wilcoxon signed-rank test was used for matched paired data, the Mann–Whitney U-test was used for comparing two independent groups, and Fisher’s exact test was used for comparing percentages of independent groups. Holm–Bonferroni correction was applied to control for false discovery rate in multiple hypothesis tests [29]. A two-sided *p*-value < 0.05 was considered statistically significant. All statistical analyses were conducted in version 22 of IBM SPSS Statistics (IBM Corp., Armonk, NY, USA) and version 4.01 of the R statistical package (R Foundation for Statistical Computing, Vienna, Austria).

### 2.6. Ethical Approval

All procedures in this study were performed in accordance with the ethical standards of the national research committee and with the 1964 Helsinki declaration. The study was approved by the regional ethical review board in Uppsala, Sweden (refs: 2005:011/1, 2017:322).

## 3. Results

### 3.1. Presence of S. aureus in CRS Patients and Antibiotic Susceptibility

**Figure 1:**
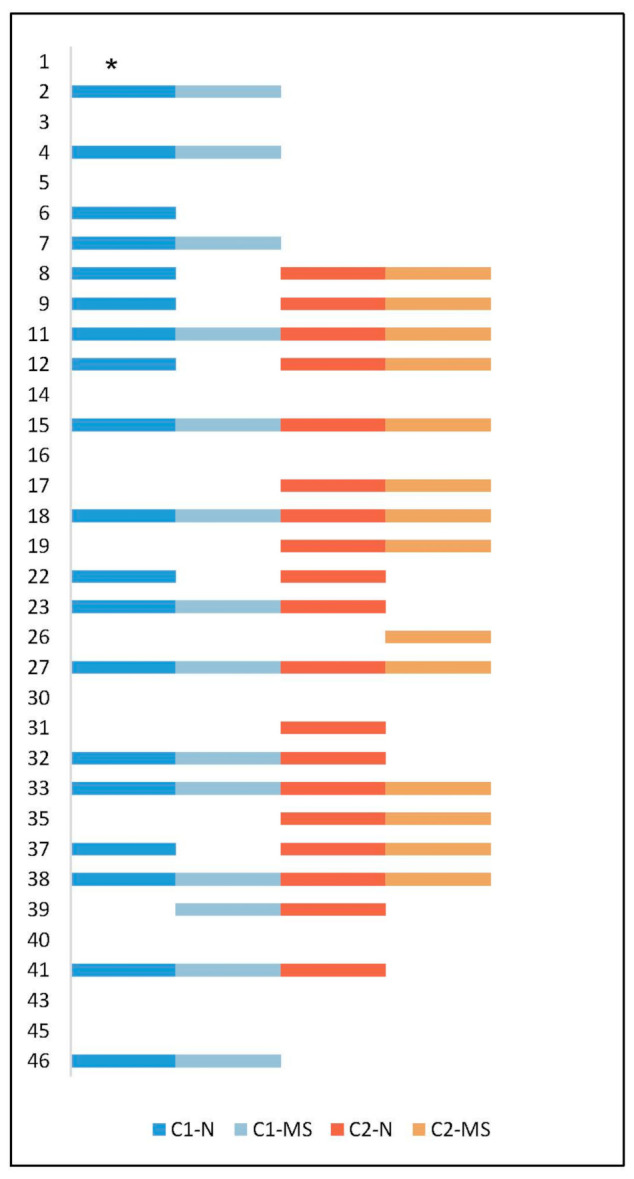
Presence of *Staphylococcus aureus* in the nares and maxillary sinus in 34 patients at initial collection (C1) and at follow-up (C2) after approximately 11 years. The Y-axis shows the study ID for all 34 patients. One step on the X-axis symbolizes *S. aureus* isolated at a specific time point and site. Sample sites: maxillary sinus (MS), nares (N). * One sample from nares at C1 was not available.

Thirty-four of 40 (85%) CRS patients could be included in the long-term follow-up (Figure 1). Of these, 14 (41%) had *S. aureus* in both the nares and maxillary sinus at initial inclusion (C1) and 13 (38%) had *S. aureus* in both locations at follow-up after approximately a decade (C2). In 15/34 (44%) patients, *S. aureus* was present in the nares and/or maxillary sinus at both initial inclusion and follow-up, and in 6/34 (18%) patients, *S. aureus* was present in both the nares and the maxillary sinus at both time points. Five of 34 (15%) patients had no *S. aureus* at C1 but had a positive culture at C2, and another 5/34 (15%) had a positive culture at C1 and a negative culture at C2 (Figure 1). Two patients had *S. aureus* only in the nares at follow-up.

The investigated *S. aureus* isolates were almost fully susceptible to all tested antibiotics. Four isolates from four different patients displayed resistance. One nasal *S. aureus* isolate (clonal complex (CC)30) obtained at C1 was resistant to tetracycline, one isolate (CC8) from maxillary sinus at C1 was resistant to cefoxitin and thus methicillin-resistant but otherwise susceptible to all tested antibiotics, one isolate (CC12) obtained from the maxillary sinus at C1 was resistant to fusidic acid, and finally one nasal isolate (CC398) at C2 was resistant to both clindamycin and erythromycin.

### 3.2. SNP-Based Phylogenetic Analysis

**Figure 2:**
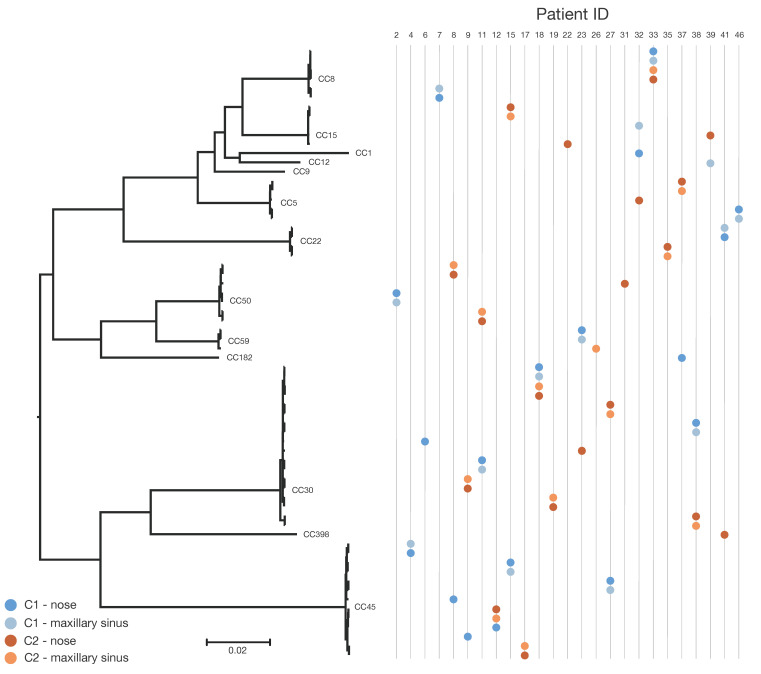
Midpoint rooted phylogenetic tree based on core genome single-nucleotide polymorphisms (SNPs) at initial collection (C1) and follow-up after 8–15 years (C2; mean follow-up: 11 years) in *S. aureus* isolated from CRS patients. Clonal complexes are presented. Based on 110,026 SNPs in the ~2.21 Mbp conserved core genome of the reference chromosome. Scale bar indicates substitutions per site. Nasal sample collected at C1 for patient number 39 was missing.

Among the 66 *S. aureus* isolates, 110,026 SNPs were identified across the conserved core genome of 77.5% (~2.21 Mbp), but generally a low number of SNPs was observed within hosts when comparing *S. aureus* isolates from the nares and maxillary sinus collected at the same time point. Figure 2 represent the relatedness of the *S. aureus* isolates based on SNP differences. All but one of the 27 paired *S. aureus* isolates collected from the nares and maxillary sinus collected at the same time point (C1 or C2) displayed the same clonal complex (CC). Three of the 15 patients with *S. aureus* in the maxillary sinus and/or nares at both time points presented identical *S. aureus* isolates (<67 SNPs over a decade). These patients (IDs 12, 18, and 33) were sampled 11, 11, and 13 years apart, respectively. Two of the three had *S. aureus* in all four samplings. The median number of SNPs for three strains with identical lineages at C1 and C2 was 48, with a range of 29–67. These persistent *S. aureus* isolates with same lineage showed no resistance to any antibiotics at either time point.

Thirteen different CCs were found and the most prevalent CCs among the isolates were CC30 (27%, 18/66) and CC45 (20%, 13/66); details are given in Table 1. These findings are illustrated using a midpoint rooted phylogeny showing the clustering of isolates according to sequence types and clonal lineages (Figure 2). Genes encoding cell-wall-associated proteins (IsaA, Plc, Efb-C, and SCIN) were present in all isolates. One isolate was lacking the gene (scn) encoding SCIN. The tst-1 gene was only present in 24% of isolates, and lukF-PV was absent from all isolates.

### 3.3. Anti-Staphylococcal Antibodies in CRS Patients

**Figure 3:**
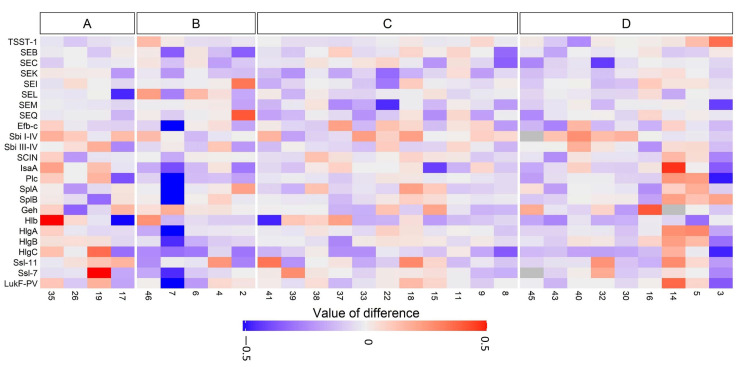
Heat map presenting changes in IgG responses toward staphylococcal antigens in serum from 29 CRS patients at initial collection (C1) and after 8–15 years (C2; mean follow-up: 11 years). Serum samples were available from 29/34 (85%) CRS patients at both time points. (**A**) Patients with *S. aureus* only at C2, (**B**) patients with *S. aureus* only at C1, (**C**) patients with *S. aureus* at both C1 and C2, and (**D**) patients with no *S. aureus* at either C1 or C2. Patient ID is indicated on the X-axis.

The humoral immune response towards 61 *S. aureus-*specific antigens was determined in sera from the 29/34 (85%) CRS patients whose serum samples were available from both time points. We focused on serum antibody responses against selected virulence factors that may be of importance for CRS, especially those directed at staphylococcal enterotoxins and microbial surface components recognizing adhesive matrix molecules (MSCRAMMs) (Figure 3). The overall change in antibody levels showed great variability over time (Figure 3). Comparing specific antibody responses between sera from the five (5/29, 17%) patients who lost *S. aureus* colonization and the four (4/29, 14%) who became colonized with *S. aureus* showed no statistically significant change in antibody levels. Nine (31%) of the 29 patients showed no *S. aureus* at either C1 or C2, while 11 (38%) showed *S. aureus* at both time points. We found no statistically significant change in anti-staphylococcal IgG in serum when comparing these two groups.

When considering the differences for anti-staphylococcal IgG in all analyzed sera, patients who lost their *S. aureus* between C1 and C2 showed significantly lower median total IgG levels than patients who became colonized with *S. aureus* at C2 (*p* = 0.03). There was no significant difference in median total IgG levels in serum from patients without *S. aureus* at C1 and C2 and those with *S. aureus* at both time points (*p* = 1.0). Two of the three patients who were regarded as persistent carriers of identical *S. aureus* strains had provided sera for analysis (patient numbers 18 and 33). Neither of them differed from the rest of the patients in terms of antibody profile.

In an effort to determine a presumably direct connection between IgG responses and specific staphylococcal antigens, we used whole-genome sequencing to assess the presence of the corresponding genes in *S. aureus* isolates from the 29 patients for whom serum was available. There was no correlation regarding presence of a specific gene in the strain and existence of anti-staphylococcal antibody response towards the corresponding staphylococcal factor in the host. We found stable IgG responses against TSST-1 over time, except for one patient with a clearly increased level at C2. All 29 CRS patients showed antibodies toward TSST-1, but the tst-1 gene was only present in 24% of the *S. aureus* isolates. These isolates were collected from five patients, and belonged to CC30 (n = 6) and CC50 (n = 1). None of the collected isolates contained lukF-PV, which contributes to encoded PVL, but 89% of the patients (25/28) showed antibodies towards this antigen. Genes encoding cell-wall-associated proteins (IsaA, Plc, and Efb-C) were present in all isolates. The genes for enterotoxins (TSST-1, SEB, SEC, SEI, SEK, SEL, SEM, and SEQ), other secreted factors (Sbi, LukF-PV, HlgA, HlgB, HlgC, Hlb, SplA, SCIN, SplB, Ssl-11, Ssl-7), and cell-wall-associated proteins (IsaA, Plc, Sbi, Efb-C) showed no correlation with IgG levels in serum.

## 4. Discussion

*S. aureus* has been suggested to have an impact on CRS, and so this long-term follow-up of CRS patients aimed to investigate persistence of *S. aureus* in the nares and maxillary sinus and to determine the humoral immune response against *S. aureus* antigens. We also used whole-genome sequencing to examine the presence of the corresponding genes in sinonasal *S. aureus*. To the best of our knowledge, no previous studies have evaluated serum IgG responses to virulence factors in CRS or performed genetic analyses of persistent *S. aureus* over such a long time span. Studies of anti-staphylococcal immune response that use isolates from clinical settings rather than laboratory strains are rare. *S. aureus* was found in the nares and/or maxillary sinus of 44% of CRS patients at initial inclusion and follow-up.

The intra-host SNP variation was very low when comparing isolates from nares and maxillary sinus collected at the same time point, and these isolates were therefore considered to originate from the same strain given the mutation rate of 2–7 SNPs/year for *S. aureus* [30]. There was one patient at C1 with a nasal isolate belonging to CC1 and a maxillary sinus isolate belonging to CC15, but otherwise these highly consistent findings indicate colonization from the nares to the maxillary sinus as one joint sinonasal milieu. We could also demonstrate a persistence of specific *S. aureus* in the nares and maxillary sinus over a decade in three of 15 (20%) CRS patients. Other studies have shown a similar proportion of persistent *S. aureus* carriers in the anterior nares (12–30% of the population), but criteria for a classification as persistent carriage are not standardized. A minimum of two cultures, often within a few weeks of each other, has been suggested as a basis for classification [6,7,31,32]. In a follow-up study from 1999, 17 persistent nasal carriers were re-investigated after eight years and only three of them could still be regarded as persistent carriers [33]. This is in accordance with the *S. aureus* persistence rate in the present study. A strength of this study is that persistence of *S. aureus* is stated by identification of CC and not just by findings of *S. aureus*. In addition, 23/34 (29%) of patients were regarded as intermittent nasal carriers due to having *S. aureus* of different CCs.

All except four isolates were susceptible to all tested antibiotics. These four were from non-persisting *S. aureus* carriers and one of these was an MRSA. The low resistance rate of the isolates in our study likely also reflects the restricted use of antibiotics in Sweden. Basic treatment for CRS symptoms comprises topical application of corticosteroids together with daily nasal saline irrigations and antibiotics [34], but in Sweden antibiotics are seldom used; in selected cases, an isoxazolyl-penicillin is preferably used as first choice. Treatment of CRS with macrolides and doxycycline is common in other countries [35], and may result in emergence of resistance.

The two major clonal complexes identified in our collection were CC30 and CC45, which represented 47% of all *S. aureus* isolates in our study. None of them showed resistance to any of the antibiotics tested. These clonal complexes are also common among the general population [36,37], and so CRS does not seem to be associated with a particular *S. aureus* genotype. Genes for cell-wall-associated proteins were highly present in the isolates. Thus, genes important for colonization were present in both persisting strains and intermittent strains. The presence of the tst-1 gene in 24% of isolates and lukF-PV in none contradicts previous results indicating these two virulence factors to be of significance in CRS [23].

We found an overall large variability in antibody levels against staphylococcal antigens. Even if the genes for enterotoxins, other secreted factors, and cell-wall-associated proteins were detected, only a weak correlation to IgG levels in serum was observed. *S. aureus* colonization of other body sites, and previous infections in particular, may be a stronger stimulator of IgG responses possessing the gene encoding effector protein.

The most frequent carriage site of *S. aureus* in humans is the anterior nares, but *S. aureus* can also be found at other body sites such as the vagina, rectum, skin, and gastrointestinal tract [7,38,39]. The anterior nares seem to function as a reservoir for carriage and hence spread, and so elimination from the nares can result in subsequent loss of the bacteria from other parts of the body [7]. This indicates the importance of the nares for carriage. A study using a twin population demonstrated that host genetics does not strongly determine the microbiota, and suggested that *S. aureus* is a major part of the nasal community in some individuals and a distinct indicator of the ecological milieu in the nose of certain patients [8].

There was a significant reduction of mean IgG toward staphylococcal antigens in sera from patients who lost *S. aureus* colonization between C1 and C2 compared to IgG levels in sera from patients displaying a positive *S. aureus* culture at follow-up. However, the lack of change of immune response in serum from patients without *S. aureus* at C1 and C2 compared to IgG levels in serum from patients with *S. aureus* at both time points provides conflicting results. Anti-staphylococcal antibody levels in serum reflect an ability to protect, to modify the course of infection, and to reduce the risk of complications of a staphylococcal infection [40,41], but our knowledge is limited regarding their role in chronic inflammatory disease. Antibody levels have previously been shown to be stable over years in healthy individuals [42]. Neutralizing serum antibodies are common but also widely variable in healthy populations [4,43,44]. However, higher IgG antibodies toward TSST-1 and SEA have been observed in persistent *S aureus* carriers than in non-carriers [40].

All of the 29 patients for whom serum and *S. aureus* isolates were available from C1 and C2 showed antibodies toward TSST-1. The tst-1 gene was only present in 24% of the *S. aureus* isolates. None of the collected isolates contained LukF-PV, encoding part of the PVL toxin, but 89% showed antibodies toward that antigen. Hence, the role of anti-staphylococcal antibodies in CRS is not obvious. A review by Holtfreter et al. concluded that antibodies against a wide range of staphylococcal enterotoxins are common in the healthy population, and increase during infection [45]. Also, Radke et al. identified a hierarchy of anti-staphylococcal proteins regarding which antigens the immune system was recognizing from *S. aureus* [46]. In addition, previous experience of staphylococcal infections such as skin and soft tissue infections with various *S. aureus* strains will probably also affect the immune response toward leucocidins and not only CRS. In addition, some individuals could be more prone to variation in antibody production. In a study by van Belkum et al., an increase in antibodies was only found in individuals with persistent nasal colonization by *S. aureus* [47]. Furthermore, it has been shown that raised antibody levels are present in *S. aureus* nasal carriers to a greater extent than in non-carriers [39]. Antibody responses in our study were not correlated to the presence of corresponding genes, indicating a previous infection or a stronger stimulator of immune response elsewhere. It would have been desirable to know the *S. aureus* carriage state at other body site as well as previous *S. aureus* infections between C1 and C2, since this could have affected the results, but this information is difficult to collect for such a long-term follow-up study. Information regarding gene expressions would also have been valuable.

## 5. Conclusions

In a Swedish cohort of CRS patients, we found decade-long persistence of *S. aureus* from the sinonasal site in 20% of our 34 patients, displaying a median of 37 SNP differences (range: 30–68). Carriage was intermittent in 35% of cases. A low number of SNPs across the core genome differed when comparing *S. aureus* from the nares and maxillary sinus collected at the same time point, indicating colonization of *S. aureus* from the nares to the maxillary sinus. The overall alterations of anti-staphylococcal antibodies over time showed great variability, and minor support for an impact of *S. aureus* on CRS. The vast majority of *S. aureus* isolates were susceptible to all tested antibiotics, including the *S. aureus* strains that had persisted for a decade.

## Figures and Tables

**Table 1:** Single-nucleotide polymorphisms (SNPs) detected between the *S. aureus* isolated at both sample sites at initial collection (C1) and/or after 8–15 years (C2; mean follow-up: 11 years) based on a conserved core genome of ~2.23 Mbp across the collection. Sample site: maxillary sinus (MS), nares (N).

Patient ID	Clonal Complex	Time Point	Sample Site	SNP Differences between C1 and C2	SNP Differences Comparing C1 to C1 or C2 to C2
2 ^c^	CC50	C1	N	Not applicable	0
2	CC50	C1	MS		
4 ^c^	CC45	C1	N	Not applicable	1
4	CC45	C1	MS		
6	CC30	C1	N	Not applicable	Not applicable
7 ^c^	CC8	C1	N	Not applicable	2
7	CC8	C1	MS		
8	CC45	C1	N	37,758–37,761	3
8	CC50	C2	N		
8	CC50	C2	MS		
9	CC45	C1	N	33,462–33,463	1
9	CC30	C2	N		
9	CC30	C2	MS		
11	CC30	C1	N	32,890–32,892	1
11	CC30	C1	MS		
11	CC50	C2	N		3
11	CC50	C2	MS		
12	CC45	C1	N	29–30 ^a^	1
12	CC45	C2	MS		
12	CC45	C2	N		
15	CC45	C1	N	41,088–41,089	1
15	CC45	C1	MS		
15	CC15	C2	N		2
15	CC15	C2	MS		
17 ^b^	CC45	C2	N	Not applicable	0
17	CC45	C2	MS		
18	CC30	C1	N	65–67 ^a^	2
18	CC30	C1	MS		
18	CC30	C2	N		0
18	CC30	C2	MS		
19 ^b^	CC30	C2	N	Not applicable	2
19	CC30	C2	MS		
22	CC9	C1	N	18,896	Not applicable
22	CC15	C2	N		
23	CC59	C1	N	32,884–32,885	3
23	CC59	C1	MS		
23	CC30	C2	N		
26	CC59	C2	MS	Not applicable	Not applicable
27	CC45	C1	N	33,500–33,505	1
27	CC45	C1	MS		
27	CC30	C2	N		4
27	CC30	C2	MS		
31	CC50	C2	N	Not applicable	Not applicable
32	CC1	C1	N	13,939–17,065	15,884
32	CC15	C1	MS		
32	CC5	C2	N		
33	CC8	C1	N	48–51 ^a^	2
33	CC8	C1	MS		
33	CC8	C2	N		5
33	CC8	C2	MS		
35 ^b^	CC22	C2	N	Not applicable	25
35	CC22	C2	MS		
37	CC182	C1	N	29,807	0
37	CC5	C2	N		
37	CC5	C2	MS		
38	CC30	C1	N	817–818	2
38	CC30	C1	MS		
38	CC30	C2	N		1
38	CC30	C2	MS		
39	CC12	C1	MS	12,182	Not applicable
39	CC15	C2	N		
41	CC22	C1	N	33,882–33,883	1
41	CC22	C1	MS		
41	CC398	C2	N		
46 ^c^	CC5	C1	N	Not applicable	1
46	CC5	C1	MS		

^a^ Considered as persistent carriage. ^b^ No growth of *S. aureus* at time of initial inclusion. ^c^ No growth of *S. aureus* at follow up.

## Data Availability

The datasets used and analyzed during the current study are available from the corresponding author on reasonable request.
